# Comparison of the Photosynthesis, Hydraulic Properties, and Anatomy of *Pteroceltis tatarinowii* Leaves Between a Limestone and a Cultivated Forest

**DOI:** 10.3390/plants13223205

**Published:** 2024-11-15

**Authors:** Ya Zhang, Yu-Die Wang, Meng-Meng Ma, Ying-Ying Zhang, Dong-Sheng Du, Xian-Can Zhu, Xiao-Hong Li

**Affiliations:** The Anhui Provincial Key Laboratory of Biodiversity Conservation and Ecological Security in the Yangtze River Basin, College of Life Sciences, Anhui Normal University, Wuhu 241000, China; zhangya@ahnu.edu.cn (Y.Z.); wyd15055273647@163.com (Y.-D.W.); 15936025558@163.com (M.-M.M.); 18298166296@163.com (Y.-Y.Z.); duds@ahnu.edu.cn (D.-S.D.); zhuxiancan@ahnu.edu.cn (X.-C.Z.)

**Keywords:** drought resistance, habitat adaption, hydraulic conductivity, photosynthesis, *Pteroceltis tatarinowii*, vein density

## Abstract

*Pteroceltis tatarinowii* Maxim is a famous paper-making tree endemic to China with a wide distribution. Leaves of this tree growing in different habitats show a certain plasticity, which is important for their ecological adaption. Here, the photosynthesis ability, hydraulic properties, and anatomy of *P. tatarinowii* leaves from a limestone forest (Langya Mountain) and a cultivated forest (Xiaoling Village) in Anhui province were compared. The results showed that leaves from Xiaoling Village had higher net photosynthesis rate and hydraulic conductivity, which were closely related to their higher vein density, stomatal density and palisade tissue thickness than leaves from Langya Mountain. However, lower leaf water potentials at turgor loss point and at 50% loss of conductivity, as well as a higher leaf hardness, for Langya Mountain leaves indicated their higher hydraulic safety and drought resistance than those of leaves from Xiaoling Village. This study reveals a hydraulic trade-off between efficiency and safety for *P. tatarinowii leaves* growing in distinct habitats. Further studies should include more habitats and different vegetation communities to clarify the ecological adaption so as to provide a scientific basis for the protection of this species.

## 1. Introduction

The frequency and intensity of drought events have gradually increased due to the continuous rise in global temperatures and changes in precipitation patterns [1]. Global forest ecosystems are also facing significant challenges, with various types of vegetation experiencing plant mortality events [2]. Among these, hydraulic failure is a major cause of plant wilting or even death [3,4]. Therefore, detecting the hydraulic architecture characteristics of plants and their self-regulating mechanisms is particularly necessary for understanding the ecological adaptability of plants [2]. Plant leaves are the hub of the connection between plants and the atmosphere, and their structural characteristics could reflect the impact of the environment on plants and the adaptability of plants to the environment [5]. Leaf hydraulic traits refer to the characteristics of survival strategies formed by leaves to adapt to the external environment in terms of water transport [6]. They limit the water transport of the entire plant, affecting the gas exchange of plants [7], and thus impact leaf economic traits as well as plant survival and growth. Leaf size, thickness, specific leaf area, and anatomical structure all have significant effects on the physiological and ecological characteristics of plants and their adaptability to the environment [8]. For example, when plants are subjected to drought stress, the stomata on the leaves tend to close in a timely manner to reduce water loss from the body, while the leaf hydraulic conductivity decreases, meanwhile suppressing photosynthesis. In addition, drought-resistant plants often have good leaf water storage capacity to buffer the limitation of leaf function from the drought events [9].

*Pteroceltis tatarinowii* Maxim, a deciduous tree from the Cannabaceae, is a unique fiber tree species native to China. The wood of this tree is hard, dense, and fine, making it a great material for high-end furniture, but, more importantly, the bark of *P. tatarinowii* is a main high-quality raw material for making the famous Xuan paper, which has been included in world intangible cultural heritage since 2006. In addition, the leaves are nutritionally rich and can be used as a nutritional feed additive [10]. *P. tatarinowii* is widely distributed in China, particularly in Anhui province where Xuan paper is originated and which harbors a rich commercial forest plantation of *P. tatarinowii*; it is also an ornamental tree in some regions, such as in Shandong Province. In the wild, *P. tatarinowii* is distributed in limestone mountain foothills, forest edges, valleys, riverbanks, stream sides, or in crevices, which makes it a good heliophyte and limestone pioneer tree example. Based on field observations, leaves of *P. tatarinowii* growing in different habitats show a variation in size and thickness which may suggest different ecological adaptions.

Research on the ecological adaption of *P. tatarinowii* includes the basic anatomical structure of the leaves [11], the photosynthetic characteristics of leaves under different environmental treatments, and the water potential of young stems and roots [12]. However, the leaf hydraulic characteristics of *P. tatarinowii* have not yet been investigated, such as leaf hydraulic conductivity and the water potential at the point of leaf turgor loss. These hydraulic traits of leaves can not only affect the efficiency of water transport and the rate of gas exchange [13], but also play a crucial role in plant growth, competition, and distribution [14,15].

**Figure 1:**
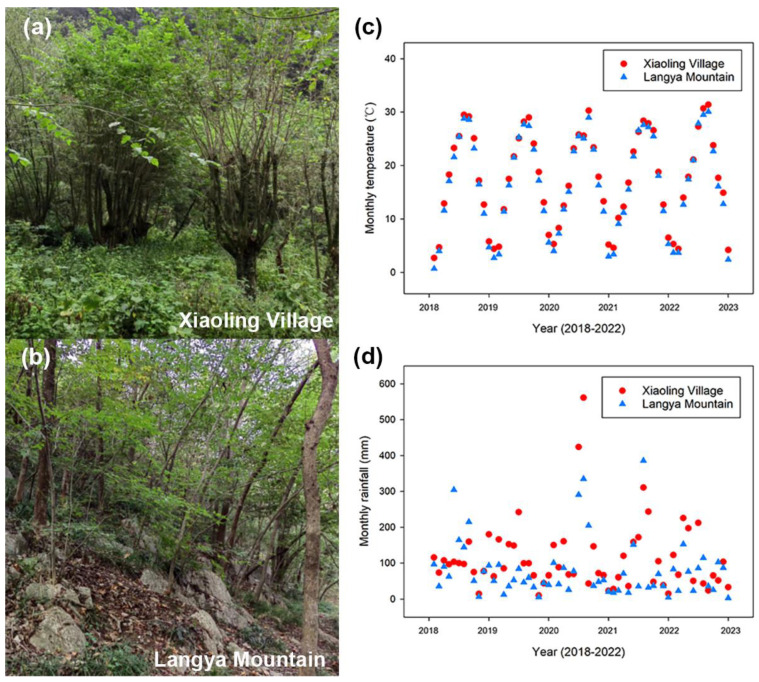
Views of two study sites and their meteorological data from 2018 to 2022. (**a**,**b**) show trees of *P. tatarinowii* growing in a cultivated forest in Xiaoling Village and a limestone forest in Langya Mountain, respectively. (**c**) and (**d**) show the monthly temperature and precipitation of the two sites, respectively. Red circles represent data of the cultivated forest in Xiaoling Village, and blue triangles represent data of the limestone forest in Langya Mountain. The meteorological data were downloaded from the Anhui Provincial Bureau of Statistics.

Here, the leaf anatomical and functional characteristics of *P. tatarinowii* growing in two different habitats in Anhui province, i.e., limestone and cultivated forests, were compared, including leaf hydraulic traits, leaf xylem anatomical structure, and photosynthetic gas exchange. Both sites are representative distribution locations of *P. tatarinowii* in Anhui Province with distinct water accessibility in natural habitats (Figure 1, see study site section). The leaves of *P. tatarinowii* from the limestone forest were expected to show lower leaf conductivity and net photosynthesis rate but higher drought resistance than those from the cultivated forest. This study helps to clarify the leaf water adaptability of *P. tatarinowii* from different habitats and provides a scientific basis for the conservation, scientific cultivation, and management of the trees.

## 2. Results

**Figure 2:**
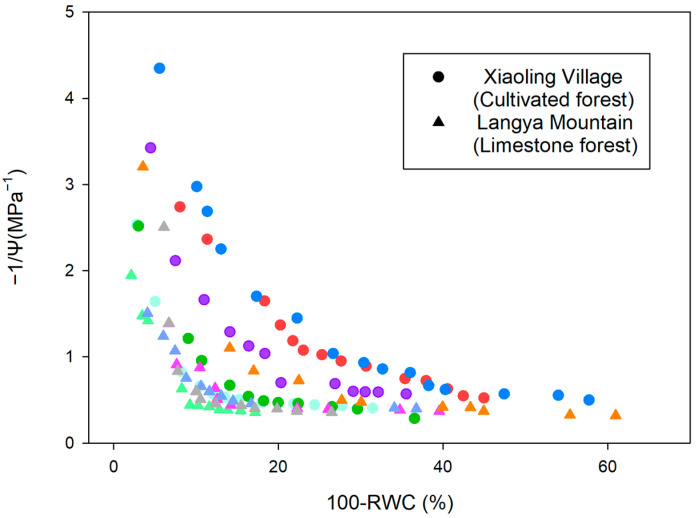
Pressure-Volume (PV) curves of *Pteroceltis tatarinowii* leaves from the two study sites. Circles and triangles represent data of leaves from the cultivated forest in Xiaoling Village and the limestone forest in Langya Mountain, respectively. Different colors represent multiple repetitions from different leaves.

**Figure 3:**
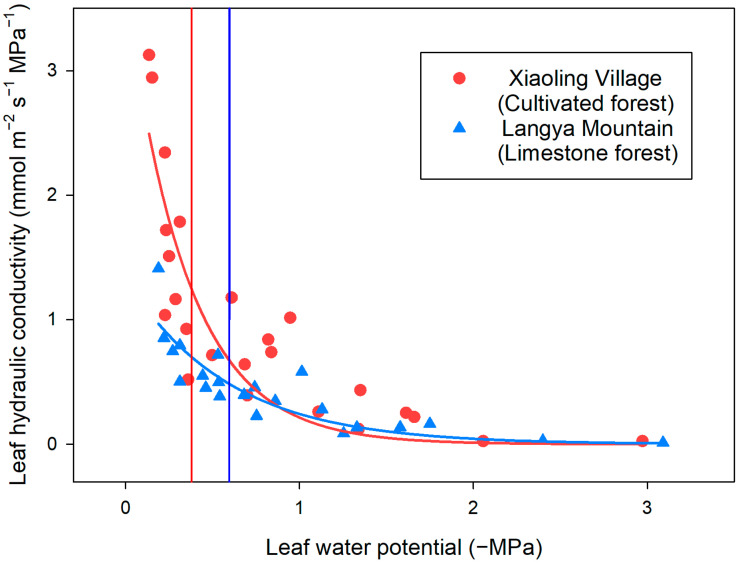
Leaf vulnerability curves of *Pteroceltis tatarinowii* from the two study sites. Red circles and blue triangles represent data of leaves from the cultivated forest in Xiaoling Village and the limestone forest in Langya Mountain, respectively. Red and blue vertical lines give the leaf water potentials at 50% loss of maximum hydraulic conductivity (P_50_) of leaves from Xiaoling Village and Langya Mountain, respectively, which were estimated from the corresponding fitted curves.

With the decrease in the leaf water potential, the leaf relative water content of *P. tatarinowii* from the two study sites decreased dramatically, but leaves from Xiaoling Village showed a faster reduction than leaves from Langya Mountain (Figure 2). Therefore, higher Ψ_TLP_ (−1.55 ± 0.55 MPa, Mean ± SD) but lower ε (5.79 ± 4.69 MPa) were found for leaves of Xiaoling Village than leaves of Langya Mountain (Ψ_TLP_ = −2.25 ± 0.11 MPa; ε = 16.29 ± 9.83 MPa) (Table 1). Higher K_max_ (2.49 mmol MPa^−1^ s^−1^ m^−2^) and Ψ_50_ (−0.38 MPa) were also found for leaves of Xiaoling Village than leaves of Langya Mountain (K_max_ = 0.97 mmol MPa^−1^ s^−1^ m^−2^; Ψ_50_ = −0.60 MPa) (Figure 3, Table 1), but the SLA of Langya Mountain leaves (324.63 ± 46.85 cm^2^ g^−1^) was higher than that of Xiaoling Village leaves (295.97 ± 32.2 cm^2^ g^−1^).

Leaves of *P. tatarinowii* from Xiaoling Village had significantly higher Pn (9.26 ± 0.98 µmol m^−2^ s^−1^) and T (2.01 ± 0.44 mol m^−2^ s^−1^) than leaves from Langya Mountain (Pn = 4.27 ± 0.77 µmol m^−2^ s^−1^; T =1.07 ± 0.37 mol m^−2^ s^−1^) (*p* < 0.01), but the latter showed higher Gs (0.3 ± 0.08 mol m^−2^ s^−1^) than the former (Gs = 0.24 ± 0.07 mol m^−2^ s^−1^) (Table 1).

In addition, *P. tatarinowii* of Xiaoling Village showed larger and thicker leaves than that of Langya Mountain, with significantly thicker upper epidermis, lower epidermis, palisade tissue, and spongy tissue (*p* < 0.05) (Table 1). However, leaves of Langya Mountain had higher PT/ST than that of Xiaoling Village (Table 1). 

**Figure 4:**
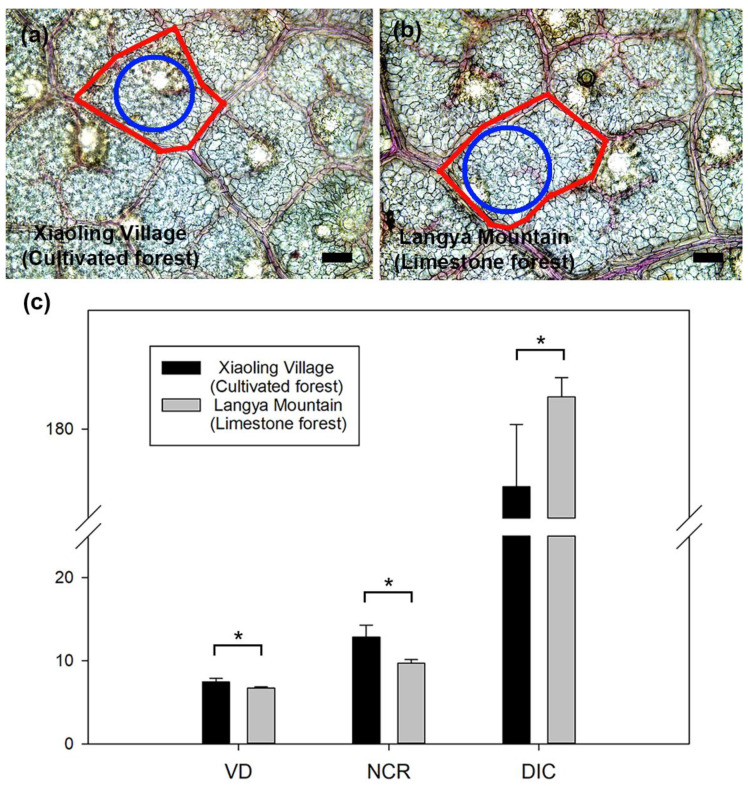
Minor veins of *Pteroceltis tatarinowii* leaves from the two study sites and their difference in vein density, number of closed vein regions, and diameter of the inscribed circle in the closed region. (**a**,**b**) show minor veins of Xiaoling Village (cultivated forest) and Langya Mountain (limestone forest) leaves, respectively. (**c**) shows significant differences between leaves of Xiaoling Village and Langya Mountain in minor vein density (VD, µm µm^−2^ × 10^−3^), number of closed vein regions (NCR, N mm^−2^), and diameter of the inscribed circle in the closed vein regions (DIC, µm). Red lines show the closed regions formed by the minor veins and blue circles represent the inscribed circles in these closed regions. Star labels indicate statistical significances (*p* < 0.05). Scale bar = 100 µm in plate (**a**,**b**).

Moreover, the stomata of *P. tatarinowii* leaves in Xiaoling Village showed a higher density (2.67 ± 1.19 N µm^−2^) but smaller size (12.51 ± 1.32 µm) than those in Langya Mountain (Table 1). Similarly, compared with leaves of Langya Mountain, the minor veins of *P. tatarinowii* leaves in Xiaoling Village showed a significant higher density (7.43 ± 0.46 µm µm^−2^ × 10^−3^) and these minor veins formed more closed regions (NCR = 12.84 ± 1.46 N mm^−2^), while when calculating the diameter of the inscribed circle in the closed regions, the leaves of Langya Mountain showed a significant higher value (DIC = 190.79 ± 6.53 µm) than those of Xiaoling Village (DIC = 160.52 ± 20.99 µm) (*p* < 0.05) (Figure 4, Table 1).

## 3. Discussion

The leaves of *P. tatarinowii* from the limestone forest and the cultivated forest showed significant differences in photosynthetic ability and hydraulic properties, which is highly supported by the leaf anatomy of the two sites, such as leaf venation. The leaf functional discrepancies of *P. tatarinowii* in the two sites indicate different water transport efficiency and safety, and suggest distinct water use strategies.

### 3.1. Leaves from the Cultivated Forest Show Higher Photosynthetic Ability than Those from the Limestone Forest Supported by Leaf Anatomy

The leaves of *P. tatarinowii* from Xiaoling Village showed significant higher net photosynthesis rate and transpiration rate than the leaves from Langya Mountain, which is in line with our hypothesis. This difference in photosynthetic properties of the leaves between the two sites is supported by the differences in their leaf anatomical traits. Firstly, the minor veins of leaves in the cultivated forest showed higher density and more closed areas than those of the limestone forest. This indicates that the leaves of Xiaoling Village may have a faster transport rate for water and photosynthetic products than those of Langya Mountain, because dense minor veins provide multiple transport pathways [16,17,18]. In addition, a short diameter of the inscribed circle in the closed areas formed by the minor veins was found in the leaves in the cultivated forest, which throws light on a short distance and less transport resistance between the mesophyll cells and minor veins [19,20,21]. This is also supported by the higher leaf hydraulic conductivity of the leaves from Xiaoling Village than those from Langya Mountain, which may contribute to the photosynthetic differences between the two sampled sites.

Secondly, leaf photosynthesis is closely related with the leaf stomata, which provide main pathways for water and carbon dioxide [22]. High stomatal density and small stomata are positively linked with a high net photosynthetic rate [23,24], which is in line with our results. Moreover, large stomata can contribute to enhance stomatal conductivity [25], which may explain the higher stomatal conductivity in the leaves of *P. tatarinowii* from the limestone forest than those from the cultivated forest. However, smaller stomata can open or close faster in response to environmental conditions and optimize water fluxes under limiting water availability [24,26,27].

Lastly, the leaves of *P. tatarinowii* from Xiaoling Village showed significant thickness with thicker palisade cells and spongy mesophyll cells, as well as a larger leaf area, than those from Langya Mountain. Large and thick leaves could have a good ability of water storage and therefore contribute to photosynthesis. Also, thick palisade cells with a large number of chloroplasts could reduce the transpiration of water and avoid the burning of mesophyll cells by strong light [28], which can effectively improve the photosynthetic rate. Nevertheless, thick leaves were also reported to show weak photosynthetic ability due to a high transport resistance of carbon dioxide and a reduced propagation of light in mesophyll tissues [29,30,31]. This disadvantage might be compensated by the high vein density in the leaves of *P. tatarinowii* in the cultivated forest.

### 3.2. Leaves from the Cultivated Forest Show a Higher Hydraulic Conductivity but Lower Hydraulic Safety than Those from the Limestone Forest

The leaves of *P. tatarinowii* from Xiaoling Village showed a significant higher maximum hydraulic conductivity than the leaves from Langya Mountain, which is consistent with the difference in venation between the two study sites. Leaf venation, stimulated by different environmental stimuli (e.g., RH%, VPD, and light) [27], is the key transport system for water and nutrients, and closely related with hydraulic efficiency and safety [15,32]. Compared with the leaves in the limestone forest, higher vein density and a smaller inscribed circle formed by the minor veins in the leaves of the cultivated forest indicate shorter transport distance, less hydraulic resistance, and higher hydraulic efficiency [19,20,21,33,34].

However, the leaves of *P. tatarinowii* from the cultivated forest showed a significantly higher P_50_ value than leaves from the limestone forest, which suggests a lower hydraulic safety. The leaf vulnerability curve characterizes the leaf hydraulic conductivity changes during the leaf water loss, i.e., the increase in leaf embolism, and the P_50_ value represents the xylem tension when the leaf hydraulic conductivity lost 50% [35,36]. The low P_50_ value for Langya Mountain leaves indicates that their xylem has a strong embolism resistance and may retain certain hydraulic function during drought events [37,38]. This is important for the leaves of *P. tatarinowii* in the limestone forest because the local precipitation is lower compared with that in the cultivated forest, which may indicate more drought events annually.

The embolism-resistant xylem in the limestone forest leaves is also supported by their PV curves which showed low Ψ_TLP_ and high ε. Ψ_TLP_ characterizes the leaf water status when turgor is lost and a low value indicates a strong embolism resistance in the leaf xylem [39,40]. Under drought stress, low Ψ_TLP_ avoids early and fast stomatal closure and reduces limitations for gas exchange during photosynthesis, which contributes to retain stomatal conductivity and hydraulic conductivity. ε is related to the hardness of the cell wall and represents the ability to resist deformation [16,41]. Leaves with a high ε value show a high mechanical strength and can resist leaf shrinkage caused by drought [42,43], which is vital to retain leaf water supply during drought events.

Moreover, compared with the leaves in the cultivated forest, the safer water transport in the leaves of *P. tatarinowii* in the limestone forest indicates better drought resistance, which is in accordance with their smaller leaf area and higher ratio of palisade to spongy tissue. Small leaves with well-developed palisade tissue are typical traits adapted to dry habitats [44,45,46]. Small blades receive less heat and have a low heat conductance, which help to reduce leaf temperature, and a higher ratio of palisade tissue could prevent damage from bright light [30,47].

## 4. Materials and Methods

### 4.1. Study Sites

The study was conducted in a limestone forest at the Langya Mountain National Forest Park in Chuzhou, Anhui Province (32°15′17″–32°21′49″ N, 118°07′35″–118°18′21″ E, elevation 321 m) and a cultivated forest in Xiaoling Village, Jingxian, Xuancheng, Anhui Province (117°57′–118°41′ E, 30°21′–30°50′ N, elevation over 300 m) from May to October 2023. Langya Mountain belongs to the karst landform of low hills and falls within the humid monsoon climate zone transitioning from the north subtropical to the warm temperate zone (Figure 1). The average annual temperature is 16.4 °C and the average annual precipitation is 985.4 mm. Although within a similar climate zone, Xiaoling Village in Jingxian has more annual precipitation with an average of 1371.9 mm. The average annual temperature is 17.4 °C, which is slightly higher than that at Langya Mountain (Figure 1). In addition, the cultivated forest at Xiaoling Village in Xuancheng is one of the main cultivation areas of *P. tatarinowii* for Xuan paper production in Anhui Province. In this forest, there is hardly any management of or interference with the trees, except for bark harvesting every three years. Therefore, both Langya Mountain and Xiaoling Village are representative distribution areas for *P. tatarinowii* in Anhui Province.

### 4.2. Pressure–Volume (PV) Curve Measurement

The pressure–volume (PV) curve characterizes the relationship between leaf water potential and leaf water content, which is established based on Tyree and Hammel [16]. In the early morning, several sunlit branches with leaves were collected from the field and quickly placed into a bucket of water. The cut ends were trimmed underwater by ca. 10 cm, and then sealed with a black plastic bag to prevent transpiration. After returning to the laboratory within 2 h, the branches were rehydrated underwater for approximately 2 h before being removed to ensure the full saturation of leaf water potential (water potential > −0.3 MPa). Five healthy leaves were randomly selected from different branches, and after wiping off surface moisture, their water potentials were measured using a pressure chamber (PMS 1505 EXP, Corvallis, OR, USA). The leaves were then quickly weighed using an analytical balance (JJ124BC, G & G, Changshu, China). The leaves were then dried naturally on the bench for different periods, during which the leaf water potential and weight were repeatedly measured until the leaves severely wilted or the leaf water potential reached ca. −3 MPa. Then, the leaves were dried at 80 °C for 48 h and weighed to obtain the dry weight. The relative leaf water content at each measurement was calculated and plotted with water potential to establish the PV curve. From the curve, the turgor loss point water potential (Ψ_TLP_, MPa), i.e., the leaf water potential when the leaf turgor is lost, was determined at the turning point between the rapidly descending part and the linear descending part of the curve. The modulus of elasticity (ε, MPa) was calculated as the ratio of changes in leaf turgor to changes in relative water content.

### 4.3. Establishment of Leaf Vulnerability Curve

The leaf vulnerability curve is determined by the transpiration method in the end of September and the beginning of October [48]. Several branches were collected and rehydrated as described above. After the rehydration, the branches were dried naturally on the bench for different periods of time. At different time intervals, one leaf from each branch was cut under water and connected to a pipette via a silicone tube filled with 10 mmol L^−1^ KCl solution. Then, the leaf was placed above a portable electric fan to enhance the transpiration. When a stable water flow was reached, the flow rate (F, g s^−1^) was calculated as the mass of water flowing through the pipette per second. Afterwards, the leaf was kept in a moist plastic bag for 30 min to maintain water equilibration. Then, the leaf water potential (Ψ, MPa) was measured using the pressure chamber and the leaf area (S, m^−2^) was determined via the ImageJ software (version 1.50i, National Institutes of Health, Bethesda, MD, USA). The leaf hydraulic conductance (K_leaf_, mmol m^−2^ s^−1^ MPa^−1^) is calculated using the following formula:

K_leaf_ = F/(Ψ × S) (1)

By repeating the above steps, a series of points between Ψ and K_leaf_ can be obtained until the leaf hydraulic conductance no longer decreases or the leaf water potential reaches ca. −3 MPa. A three-parameter sigmoidal model was fitted to the data in SigmaPlot14.0 (SPSS Inc., Chicago, IL, USA) to obtain the leaf vulnerability curve. From the curve, the maximum leaf hydraulic conductivity (K_max_, mmol m^−2^ s^−1^ MPa^−1^) was estimated when the leaf water potential approached 0. Then, the water potential at 50% of the maximum leaf hydraulic conductivity was estimated for Ψ_50_ (MPa).

### 4.4. Measurement of Leaf Relative Water Content and Specific Leaf Area

Fresh branches were collected and brought back to the laboratory as described above. From the branches, six healthy leaves were randomly selected and weighed using the analytical balance to obtain the fresh weight. The leaves were then completely submerged in deionized water in the dark for 24 h to obtain the saturated fresh weight. The leaf area (S, cm^2^) was then measured using ImageJ software (version 1.50i). Finally, the leaves were placed in a 72 °C oven and dried for 48 h to obtain the dry weight. The relative water content (RWC, %) of the leaves was calculated as the ratio of the difference between fresh and dry weight to the difference between saturated fresh and dry weight. The specific leaf area (SLA, cm^2^ g^−1^) was the leaf area divided by the dry weight.

### 4.5. Measurement of Veins and Stomata

For the measurements of veins, 8–10 mature leaves were selected from each study site and a ca. 2 cm^2^ piece from the middle of each leaf was cut. These pieces were boiled in a 5% NaOH solution for 10 min, washed with water, carefully cleaned with a paintbrush, and observed under a light microscope (DMi8, Leica, Wetzlar, Germany). More than 20 images were taken and opened in ImageJ software (version 1.50i). An area of interest (AOI) was selected to measure the minor vein density (VD, µm µm^−2^), which is the ratio of the total length of the minor vein in AOI to the area of AOI. In addition, the number of closed regions formed by the minor veins per AOI (NCR, N mm^−2^) and the diameter of the inscribed circle in each closed region (DIC, µm) underwent at least 30 measurements at each study site.

For the measurement of stomata, avoiding the main vein, an area of ca. 1 cm^2^ in the middle of the back surface of the leaf was selected and nail polish (Rose About, Yiwu, China) was applied to it. Once dried, the nail polish was taken off and observed under the light microscope. More than 30 images of stomata were taken and opened in ImageJ to measure the length of the guard cells and the stomatal density, which is the number of stomata per leaf area.

### 4.6. Measurement of Leaf Cross-Sectional Structure

For each study site, 8–10 mature leaves were selected and a ca. 1 cm^2^ piece from the middle of each leaf was cut. The cross-sections of the piece with a thickness of 25–30 μm were obtained using a rotary microtome (Leica RM2016, Shanghai, China). Then, sections were stained with a mixture of 1% safranin and 0.5% fast blue for 5–10 min. After staining, the sections were rinsed with water and dehydrated using a gradient alcohol solution (50%, 70%, 85%, 90%, 95%, and 100%) for 1 min each. Then, the sections were transferred onto slides, one drop of neutral resin was added, and the sections were observed under the light microscope. More than 60 images were taken and opened in ImageJ to measure the thickness of the upper epidermis, palisade tissue, spongy tissue, lower epidermis, and the total thickness of the leaf. Then, the ratio of the palisade tissue to the spongy tissue thickness was calculated, as well as the ratio of the palisade tissue thickness to the leaf thickness.

### 4.7. Measurement of Photosynthetic Parameters

The photosynthetic gas exchange parameters were measured in the morning between 9:00 and 11:00 in the end of September and the beginning of October using the LI-6400 portable photosynthesis system (LI-COR, Lincoln, Dearborn, MI, USA). During the measurement, the ambient atmospheric CO_2_ concentration was applied, which is about 500 µmol mol^−1^. The effective photosynthetic radiation was set at 1200 µmol mol^−1^. At each study site, at least 5 leaves were measured for the net photosynthetic rate (Pn, µmol m^−2^ s^−1^), stomatal conductance (Gs, mol m^−2^ s^−1^), and transpiration rate (T, mol m^−2^ s^−1^).

### 4.8. Statistics

Leaf hydraulic characteristics, leaf anatomical traits, and leaf photosynthetic parameters of *P. tatarinowii* from the two study sites were compared using the Independent Samples *t*-tests in SPSS software (version 25.0, SAS Institute Inc., Cary, NC, USA). For each trait, all measurements with five duplications at each site were summarized and further averaged to obtain means for statistical analyses, except for leaf P_50_ and K_max_ values since both traits only show one single value when obtained from the vulnerability curve. In the test, the homogeneity of variances was checked first using the Levene’s Test, and then a significant difference for a specific trait was determined when *p* < 0.05. Graphs were made in SigmaPlot 14.0 (Systat Software Inc., Düsseldorf, Germany).

## 5. Conclusions

Leaves of *P. tatarinowii* from two study sites show significant differences in anatomical and functional traits. The leaves from the cultivated forest have high thickness, stomatal density, and vein density, which lead to high photosynthetic ability and hydraulic conductivity. However, the leaves from the limestone forest show low Ψ_TLP_ and P_50_, but high ε and palisade tissue ratio, which suggest high hydraulic safety and drought resistance. The leaf hydraulic properties of *P. tatarinowii* from the two habitats reveal a trade-off between water transport efficiency and safety in leaf xylem, which may help to understand the different hydraulic strategies of this species. However, considering the wide distribution of *P. tatarinowii* in China, further studies are needed, including more distinct habitats, to clarify the ecological adaptations of *P. tatarinowii*.

## Figures and Tables

**Table 1:** Comparisons of leaf hydraulic, photosynthesis, and anatomical traits (Mean ± SD) of *Pteroceltis tatarinowii* between Xiaoling Village (cultivated forest) and Langya Mountain (limestone forest) with statistical values.

Traits	Abbreviation (Unit)	Xiaoling Village (Cultivated Forest)	Langya Mountain (Limestone Forest)	F-Value	t-Value	*p*-Value
Turgor loss point water potential *	ΨTLP (MPa)	−1.55 ± 0.55	−2.25 ± 0.11	0.090	−3.352	0.011
Modulus of elasticity *	ε (MPa)	5.79 ± 4.69	16.29 ± 9.83	2.313	−3.025	0.016
Maximum hydraulic conductivity	K_max_ (mmol MPa^−1^ s^−1^ m^−2^)	2.49	0.97	/	/	/
Leaf water potential at 50% loss of maximum hydraulic conductivity	Ψ_50_ (MPa)	−0.38	−0.60	/	/	/
Net photosynthesis ***	Pn (µmol m^−2^ s^−1^)	9.26 ± 0.98	4.27 ± 0.77	1.345	8.941	<0.001
Stomatal conductivity	Gs (mol m^−2^ s^−1^)	0.24 ± 0.07	0.3 ± 0.08	0.146	−1.267	0.241
Transpiration rate **	T (mol m^−2^ s^−1^)	2.01 ± 0.44	1.07 ± 0.37	0.003	3.666	0.006
Leaf area	S (cm^2^)	52.05 ± 5.39	48.89 ± 4.03	0.276	−1.05	0.324
Specific leaf area **	SLA (cm^2^ g^−1^)	295.97 ± 32.2	324.63 ± 46.85	7.583	−4.871	0.001
Relative water content *	RWC (%)	90.23 ± 3.33	84.11 ± 3.13	0.084	2.997	0.017
Leaf thickness **	LT (µm)	179.5 ± 19.85	115.78 ± 19.5	0.022	5.119	0.001
Palisade tissue thickness **	PT (µm)	60.58 ± 9.17	40.33 ± 5.09	2.479	4.316	0.003
Sponge tissue thickness **	ST (µm)	81.02 ± 12.72	49.62 ± 12.19	<0.001	3.986	0.004
Upper epidermis thickness *	UE (µm)	26.8 ± 4.9	17.68 ± 4.4	0.059	3.100	0.015
Lower epidermis thickness **	LE (µm)	23.53 ± 3.17	17.19 ± 2.25	0.619	3.645	0.007
Palisade tissue thickness/leaf thickness	PT/LT	0.34 ± 0.02	0.35 ± 0.03	0.912	−1.015	0.340
Palisade/sponge tissue thickness	PT/ST	0.77 ± 0.03	0.86 ± 0.15	14.495	−1.398	0.230
Stomatal density	SD (N µm^−2^)	2.67 ± 1.19	1.57 ± 0.07	47.326	2.059	0.108
Stomatal length *	SL (µm)	12.51 ± 1.32	14.87 ± 0.92	0.029	−3.279	0.011
Minor vein density *	VD (µm µm^−2^ × 10^−3^)	7.43 ± 0.46	6.72 ± 0.15	7.188	3.303	0.023
Number of closed vein regions **	NCR (N mm^−2^)	12.84 ± 1.46	9.68 ± 0.46	2.321	4.601	0.002
Diameter of the inscribed circle in the closed vein region *	DIC (µm)	160.52 ± 20.99	190.79 ± 6.53	10.165	−3.078	0.029

Note: Each trait with five measurements for both sites were compared using *t*-tests, except for K_max_ and P_50_ since both traits only show one single value when obtained from the vulnerability curve. *, **, and *** indicate significant differences with *p* < 0.05, *p* < 0.01, and *p* < 0.001, respectively.

## Data Availability

Data are contained within the article.
